# Predictors of cigarette discontinuation and reduction among adults never planning to quit smoking: Longitudinal findings from the U.S. Population Assessment of Tobacco and Health (PATH) study, 2014/15–2021

**DOI:** 10.18332/tpc/225322

**Published:** 2026-09-30

**Authors:** Karin A. Kasza, Martin C. Mahoney, Andrew Anesetti-Rothermel, Heather L. Kimmel, Eva Sharma, K. Michael Cummings, Adam F. Benson, Willa Dong, Kathryn C. Edwards, Daniela Marshall, Lauren R. Pacek, Jacqueline Reuben, Mark Rinella, Candrea Smith, Wondimu Teka, Dorothy Hatsukami, Andrew Hyland

**Affiliations:** 1 Department of Health Behavior Roswell Park Comprehensive Cancer Center Buffalo United States; 2 Office of Science, Center for Tobacco Products, Food and Drug Administration Silver Spring United States; 3 Division of Epidemiology, Services and Prevention Research, National Institute on Drug Abuse, National Institutes of Health Bethesda United States; 4 Public Health, Westat Rockville United States; 5 Department of Psychiatry and Behavioral Sciences Medical University of South Carolina Charleston United States; 6 Axle Informatics Rockville United States; 7 Department of Psychiatry and Behavioral Sciences Masonic Cancer Center, University of Minnesota Minneapolis United States

**Keywords:** population, cessation, cigarettes, e-cigarettes, intentions

## Abstract

**Introduction:**

E-cigarettes have increased in use prevalence among adults who smoke cigarettes. However, the population-based literature on e-cigarette use and smoking cessation largely still focuses on those planning or attempting to quit smoking. We evaluated predictors of cigarette discontinuation or reduction among adults not planning to ever quit smoking.

**Methods:**

We conducted secondary data analyses using the Population Assessment of Tobacco and Health (PATH) Study (2014–2021; Waves 2–6) cohort data collected in the United States. Data were collected using audio computer-assisted in-person or telephone self-interviews. Participants were aged ≥21 years who smoked cigarettes every day, did not plan to ever quit smoking, and did not use e-cigarettes at baseline assessment. Predictors were previous quit attempt, quitting interest, nicotine dependence, cigarette pack-years, e-cigarette uptake (none, non-daily, daily uptake), e-cigarette flavor (tobacco, menthol/mint, fruit/sweet/other, combination flavors), e-cigarette device type (disposable, cartridge, tank/mod). Outcomes were cigarette discontinuation (from daily to no smoking) and cigarette reduction (from daily to non-daily smoking) among those who did not discontinue smoking. Weighted and adjusted generalized estimating equation logistic regression analyses were conducted.

**Results:**

Assessment in 2018/19–2021 (vs 2014/15–2015/16) was associated with higher odds of cigarette reduction (adjusted odds ratio, AOR=2.32; 95% CI: 1.01–5.36). Higher nicotine dependence (vs lower) was associated with lower odds of cigarette reduction (2.3% vs 6.7%, AOR=0.48; 95% CI: 0.25–0.90). Uptake of daily e-cigarette use (vs no uptake) was associated with reducing or discontinuing cigarette smoking (53.5% vs 10.0%, AOR=14.58; 95% CI: 5.99–35.50); the rate of daily e-cigarette uptake was 2%.

**Conclusions:**

Uptake of daily e-cigarette use was associated with cigarette reduction/discontinuation among people who smoked daily and did not plan to ever quit. Findings add to growing evidence on the role of e-cigarettes in moving adults who smoke down the continuum of risk, particularly among an understudied population at high risk of smoking-attributable morbidity and mortality.

## Introduction

It has been over twenty years since the publication of National Cancer Institute Monograph 15, ‘Those who continue to smoke: is achieving abstinence harder and do we need to change our interventions?’^1^. Despite major declines in cigarette smoking prevalence in the United States (US) over the past several decades^2^, among adults aged ≥18 years in the US today who smoke cigarettes, 17% smoke them every day and do not plan to ever quit smoking in their lifetime^3^. This proportion of people with no plans to ever quit is similar to the proportion of people who smoked cigarettes daily and had never made a quit attempt at the turn of the past century^4^. Adults who smoke and have no plans to ever quit tend to be older (aged ≥40 years), non-Hispanic White, smoke more heavily (≥20 cigarettes per day), and have lower level of education and income than their counterparts who do plan to quit smoking^3^. Therefore, persistent high rates of smoking-attributable morbidity and mortality outcomes due to cigarette smoking may be perpetuated or exacerbated without effective cessation interventions for people with no plans to ever quit smoking^1^.

During the past decade, e-cigarettes have increased in use prevalence, including among adults who smoke cigarettes^5^. However, the population-based literature on e-cigarette use and smoking cessation largely still focuses on those who are already planning or attempting to quit smoking^6^. One exception is a 2014–2019 study of adults aged ≥18 years who smoked cigarettes and did not plan to quit, which found that those who took up daily e-cigarette use experienced over eight-fold higher odds of discontinuing cigarette smoking, compared to their counterparts who did not take up any e-cigarette use (28% vs 6% cigarette discontinuation rates)^3^. These population findings are consistent with experimental studies that have shown that providing e-cigarettes to those who do not intend to quit smoking is positively associated with reductions in cigarette smoking, including quitting^7-9^.

Beyond not planning to ever quit smoking, additional factors, such as having little/no interest in quitting or having high nicotine dependence, may further diminish one’s chances of quitting, and daily e-cigarette uptake among these persons may have a different association with quitting than the association between daily e-cigarette uptake and quitting among those with some interest in quitting or those with low nicotine dependence^10-13^. Additionally, the previous population study^3^ among those not planning to quit was limited to data collected up to 2019. Since then, the e-cigarette market has continued to expand^5,14^, various tobacco control actions have been taken in the US^15-17^, and the COVID-19 pandemic occurred, any or all of which may have impacted the relationship between e-cigarette uptake and transitions in cigarette smoking among people who smoked cigarettes and had no plans to quit.

The objectives of this study were to: 1) determine the characteristics of adults aged ≥21 years in the US who smoked cigarettes daily and did not plan to ever quit smoking; 2) investigate whether and how cigarette smoking-related measures (previous quit attempt, interest in quitting, dependence, pack-years), sociodemographic characteristics, and historical time-period relate to cigarette discontinuation, and whether and how these measures relate to cigarette reduction among those who did not discontinue smoking; 3) evaluate the association between e-cigarette uptake and cigarette discontinuation or reduction; and 4) among those who took up e-cigarette use, to evaluate the association between e-cigarette characteristics and cigarette discontinuation or reduction.

## Methods

### Participants

We conducted a secondary data analysis using data from adults aged ≥21 years who participated in the nationally representative and longitudinal Population Assessment of Tobacco and Health (PATH) Study, which has been collecting data on tobacco use behaviors in the US since 2013 and is ongoing^18^. We limited analyses to those aged ≥21 years to align with the age for legal sale of tobacco products under Tobacco 21 laws, which were implemented in various states at various times over the course of the study period and which became federal law in December 2019^17^. The data analyzed here were collected using audio computer-assisted self-interviews (ACASI) conducted in-person in English or Spanish from October 2014–October 2015 (Wave 2, [W2] referred to as 2014/15), October 2015–October 2016 (Wave 3, [W3] referred to as 2015/16), December 2016–January 2018 (Wave 4, [W4] referred to as 2016/17), and December 2018–November 2019 (Wave 5, [W5] referred to as 2018/19). We also used data collected using ACASI in-person interviews and telephone interviews from March 2021 to November 2021 (Wave 6, [W6] referred to as 2021)^18^.

The PATH Study used a stratified address-based, area-probability sampling design that oversampled adults who use tobacco, those aged 18–24 years, and African American adults in Wave 1 (W1 : 2013/14). An in-person screener was used to randomly select individuals from households for participation in the study. The PATH Study was conducted by Westat and approved by the Westat Institutional Review Board. All adult participants aged ≥18 years provided informed consent. The overall weighted response rate for adults in the W1 Cohort was 83.2% in W2, 78.4% in W3, 73.5% in W4, 69.4% in W5, and 57.5% in W6^18^. Further details regarding the PATH Study design and methods^19-21^ and demographic and tobacco use distributions^22^ are published elsewhere. Details on interviewing procedures, questionnaires, sampling, weighting, response rates, and accessing PATH Study Restricted Use Files are available online^18^.

Data from Wave 1 of the PATH Study were not included in analyses so that the items used to assess e-cigarette use across waves were more comparable (e.g. e-cigarette flavor categories). Data were analyzed as approximately annual or biennial wave pairs with the analytic sample composed of adults who at the baseline wave of a given wave pair were aged ≥21 years, smoked cigarettes every day, did not plan to ever quit smoking, did not currently use e-cigarettes, and had e-cigarette uptake and outcome measure data collected at the following wave of the given wave pair (i.e. W2-W3, W3-W4, W4-W5, W5-W6; n=2353 observations and n=1309 individuals for cigarette discontinuation analyses; n=2223 observations and n=1228 individuals for cigarette reduction analyses).

### Measures

Respondents were asked about their cigarette smoking and e-cigarette use behaviors, their sociodemographic characteristics, and their health histories. Supplementary file Table 1 shows measures of the sample collected at baseline, including sociodemographic characteristics, health status, smoking-related measures, and interview mode, which were considered as potential confounders, measures used to define the sample at baseline wave, measures of e-cigarette uptake, and cigarette discontinuation (daily smoking to no smoking) and cigarette reduction (daily smoking to non-daily smoking among those who did not discontinue smoking). Supplementary file Table 1 also indicates the relative timing of assessment of each measure (i.e. at baseline wave or at follow-up wave of each wave pair, further described in the statistical analysis section).

### Statistical analysis

First, using wave pairs as described above, we described characteristics of the US population of adults who at baseline wave were aged ≥21 years, smoked cigarettes daily, had no plans to ever quit smoking, and did not use e-cigarettes, using frequencies and means for age. Next, we determined rates of cigarette discontinuation and rates of cigarette reduction at follow-up, overall and by smoking-related measures, sociodemographic characteristics, diagnosis of heart condition, diagnosis of lung condition, time-period, and interview mode. Unadjusted rates were generated and weighted to generate nationally representative estimates. Next, we used generalized estimating equations (GEE) logistic regression analyses to evaluate whether cigarette discontinuation rates, and separately whether cigarette reduction rates, differed among categories for each smoking-related measure, each sociodemographic characteristic, diagnosis of heart condition, diagnosis of lung condition, time-period, and interview mode, weighted and adjusted for all other variables as indicated in the results.

Additionally, we determined rates of cigarette discontinuation or reduction at follow-up, by e-cigarette uptake (weighted and unadjusted as above). Here, we used the combined cigarette discontinuation or reduction outcome due to small sample sizes of the e-cigarette uptake groups. We used GEE logistic regression analyses to evaluate whether cigarette discontinuation or reduction rates differed as a function of e-cigarette uptake, weighted and adjusted for age, sex, race/ethnicity, income, Heaviness of Smoking Index (HSI, a measure of nicotine dependence)^23^, time-period, and interview mode. Then, among those who took up e-cigarette use, we determined rates of cigarette discontinuation or reduction at follow-up by e-cigarette flavor, e-cigarette device type, and e-cigarette use frequency (weighted and unadjusted as above). We used GEE logistic regression analyses to evaluate whether rates differed as a function of e-cigarette flavor, e-cigarette device type, and e-cigarette use frequency, weighted and adjusted for age, sex, race/ethnicity, income, HSI, time-period, interview mode, e-cigarette flavor, e-cigarette device type, and e-cigarette use frequency.

Last, we used GEE logistic regression analyses to evaluate whether smoking-related measures, sociodemographic characteristics, diagnosis of heart condition, diagnosis of lung condition, time-period, and interview mode were associated with uptake of daily e-cigarette use, weighted and adjusted, missing data were handled using listwise deletion. Analysis-specific details are provided in the table footnotes.

GEE allows for inclusion of multiple wave pair observations in a single analysis while statistically controlling for interdependence among observations contributed by the same individuals^24,25^. We specified the unstructured covariance and within-person correlation matrices and the binomial distribution of the dependent variable using the logit link function. Analyses were run on the W2–W6 Restricted Use Files (available online)^18^, using Stata V16 software.

Estimates were weighted using the W6 all-waves weights for the W1 Cohort to adjust for the PATH Study’s complex study design characteristics (e.g. oversampling) and attrition, making them representative of the resident (excluding those institutionalized and those serving in the military) adult population of the US at the time of W6 (2021), who were also in the civilian non-institutionalized population at the time of W1 (2013/14)^18^. Variances were computed using the balanced repeated replication method^26^ with Fay’s adjustment set to 0.3 to increase estimate stability^27^. The logit method was used to calculate 95% confidence intervals (CIs). A two-tailed alpha level of 0.05 was used to determine statistical significance.

## Results

### Characteristics of the population

The population of adults in the US aged ≥21 years who smoke cigarettes daily, have no plans to ever quit smoking, and do not use e-cigarettes (n=2353 observations from n=1309 individuals) were 54.7% (95% CI: 51.4–57.9) male, 45.3% (95% CI: 42.1–48.6) female; 75.3% (95% CI: 72.1–78.1) Non-Hispanic White, 10.7% (95% CI: 9.1–12.6) Non-Hispanic Black, 3.9% (95% CI: 2.9–5.3) Non-Hispanic other race including multi-racial; 10.1% (95% CI: 8.0–12.7) Hispanic; 4.8% (95% CI: 3.9–5.8) aged 21–24 years, 24.7% (95% CI: 22.2–27.4) aged 25–39 years, 31.0% (95% CI: 27.4–34.9) aged 40–54 years, 31.1% (95% CI: 27.9–34.5) aged 55–69 years, and 8.4% (95% CI: 6.6–10.7) aged ≥70 years (Table 1). Nearly a quarter (24.3%; 95% CI: 20.8–28.0) had been diagnosed with a heart or lung condition, and over half (54.8%; 95% CI: 51.4–58.2) had ≥20 pack-years of cigarette smoking (Table 1).

**Table 1:** Characteristics of adults in the United States aged ≥21 years who smoke cigarettes daily, have no plans to ever quit smoking, and do not use e-cigarettes as of the baseline wave, Population Assessment of Tobacco and Health (PATH) study, 2014/15–2021 (N=2353 observations from N=1309 individuals)

Characteristics	Categories	n	%	95% **CI**
**Age** (years)	21–24	156	4.8	3.9–5.8
	25–39	595	24.7	22.2–27.4
	40–54	740	31.0	27.4–34.9
	55–69	700	31.1	27.9–34.5
	≥70	162	8.4	6.6–10.7
	Mean	2353	49.2	48.3–50.2
**Sex**	Male	1094	54.7	51.4–57.9
	Female	1259	45.3	42.1–48.6
**Race/Ethnicity**	Non-Hispanic White	1650	75.3	72.1–78.1
	Non-Hispanic Black	322	10.7	9.1–12.6
	Non-Hispanic, other race including multi-racial	121	3.9	2.9–5.3
	Hispanic	260	10.1	8.0–12.7
**Household income** ($)	<75000	2073	86.7	84.1–88.9
	≥75000	159	7.9	6.2–9.8
	Not reported	121	5.5	4.1–7.2
**Education level**	Lower than high school/GED	900	36.5	33.5–39.6
	High school graduate	730	33.6	30.5–36.9
	Some college/associate	584	23.5	20.3–26.9
	Bachelor’s degree or higher	139	6.4	4.7–8.7
**Ever diagnosed with heart condition** ^**a**^	No	2134	90.4	87.8–92.5
	Yes	219	9.6	7.5–12.2
**Ever diagnosed with lung condition** ^**b**^	No	1868	80.6	77.3–83.6
	Yes	485	19.4	16.4–22.7
**Ever diagnosed with heart or lung condition** ^**c**^	No	1764	75.7	72.0–79.2
	Yes	589	24.3	20.8–28.0
**Past year quit attempt**	No	2139	91.7	90.5–92.8
	Yes	214	8.3	7.2–9.5
**Interest in quitting**	None	771	32.1	29.5–34.8
	Low	784	34.3	31.6–37.1
	Moderate/high	798	33.7	30.7–36.7
**Perceived addiction**	Not at all addicted	242	10.0	8.1–12.2
	Somewhat addicted	994	42.3	39.1–45.6
	Very addicted	1117	47.7	44.3–51.2
**Heaviness of Smoking Index** (HSI)	0–3	1568	65.2	61.8–68.5
	4–6	785	34.8	31.5–38.2
**Pack-years**	<20	1113	45.2	41.8–48.6
	≥20	1240	54.8	51.4–58.2
**Time-period of PATH Study**	2014/15–2015/16 (W2-W3)	470	19.3	17.9–20.9
	2015/16–2016/17 (W3-W4)	554	23.6	22.3–24.9
	2016/17–2018/19 (W4-W5)	654	28.8	27.3–30.3
	2018/19–2021 (W5-W6)	675	28.3	27.0–29.7
**Interview mode** ^**d**^	In-person	1957	85.1	83.4–86.6
	Telephone	396	14.9	13.4–16.6

Data were aggregated across the study period and analyzed as wave pairs. Sample sizes (n) are unweighted; Percentages, 95% CIs and mean are weighted. PATH: Population Assessment of Tobacco and Health. W: Wave. GED: Generalized Equivalency Diploma. ACASI: audio computer-assisted self-interview.

a Ever diagnosed with congestive heart failure, a stroke, or a heart attack.

b Ever diagnosed with chronic obstructive pulmonary disease, chronic bronchitis, or emphysema.

c Ever diagnosed with congestive heart failure, a stroke, a heart attack, chronic obstructive pulmonary disease, chronic bronchitis, or emphysema.

d Interview mode variable from follow-up wave was used rather than from baseline wave due to W6 data collection using both ACASI in-person interviews and telephone interviews.

### Predictors of cigarette discontinuation among adults with no plans to ever quit

Among adults who at baseline were aged ≥21 years, smoked cigarettes every day, did not use e-cigarettes, and had no plans to ever quit cigarette smoking, 6.0% (95% CI: 4.5–7.6) discontinued cigarette smoking at follow-up (Table 2). The only measures associated with cigarette discontinuation were: income where those with household income ($) of ≥75000 had three-fold higher odds of discontinuing cigarette smoking than those with income <75000 (12.4% vs 5.4%, adjusted odds ratio, AOR=3.04; 95% CI: 1.17–7.93) (Table 2); and pack-years where those with ≥20 pack-years had less than one-half the odds of discontinuing cigarette smoking than those with <20 pack-years of smoking (4.6% vs 7.7%, AOR=0.45; 95% CI: 0.21–0.97) (Table 2).

**Table 2:** Predictors of cigarette discontinuation* among adults in the United States who at baseline were aged ≥21 years, smoked cigarettes daily, had no plans to ever quit smoking, and did not use e-cigarettes, Population Assessment of Tobacco and Health (PATH) study, 2014/15–2021 (N=2353 observations from N=1309 individuals)

Characteristics		%	95% **CI**	AOR	95% **CI**
**Overall**		6.0	4.7–7.6		
**Age** (years)	21–24	9.4	5.7–15.2	1.03	0.36–2.99
	25–39	5.5	3.6–8.4	0.62	0.27–1.46
	40–54	4.8	3.2–7.3	0.79	0.39–1.60
	55–69 (ref.)	5.8	3.8–9.0	1	
	≥70	10.3	4.0–24.1	1.82	0.56–5.86
**Sex**	Male (ref.)	6.2	4.8–8.0	1	
	Female	5.8	3.8–8.6	0.84	0.50–1.41
**Race/ethnicity**	Non-Hispanic White (ref.)	6.0	4.5–8.0	1	
	Non-Hispanic Black	4.1	2.1–8.1	0.62	0.26–1.53
	Non-Hispanic, other race including multi-racial	5.0	2.3–10.5	0.73	0.28–1.88
	Hispanic	8.3	5.2–12.9	1.24	0.69–2.24
**Household income **($)	<75000 (ref.)	5.4	4.2–7.1	1	
	≥75000	12.4	6.9–21.4	**3.04**	**1.17–7.93**
	Not reported	5.8	2.3–13.7	0.89	0.25–3.19
**Education level**	Lower than high school/GED (ref.)	5.2	3.7–7.4	1	
	High School graduate	6.3	3.9–10.1	1.13	0.61–2.08
	Some college/associate	6.9	4.8–9.9	1.20	0.68–2.11
	Bachelor’s degree or higher	5.5	1.6–17.4	0.50	0.10–2.57
**Ever diagnosed with heart condition** ^a^	No (ref.)	5.6	4.3–7.3	1	
	Yes	9.4	5.2–16.5	2.17	0.96–4.93
**Ever diagnosed with lung condition** ^b^	No (ref.)	6.1	4.7–7.9	1	
	Yes	5.7	3.6–9.0	1.08	0.57–2.02
**Ever diagnosed with heart or lung condition^c^**	No	5.7	4.3–7.5	^#^	
	Yes	6.9	4.5–10.3		
**Past year quit attempt**	No (ref.)	6.2	4.8–7.9	1	
	Yes	4.0	1.5–10.6	0.59	0.18–1.91
**Interest in quitting**	None (ref.)	7.0	4.6–10.4	1	
	Low	5.4	3.7–7.8	0.78	0.46–1.34
	Moderate/high	5.7	3.9–8.2	0.87	0.51–1.47
**Perceived addiction**	Not at all addicted (ref.)	6.8	3.4–13.3	1	
	Somewhat addicted	6.4	4.4–9.1	1.00	0.44–2.27
	Very addicted	5.5	4.1–7.4	1.01	0.41–2.46
**Heaviness of Smoking Index **(HSI)	0–3 (ref.)	7.0	5.3–9.1	1	
	4–6	4.2	2.8–6.1	0.68	0.38–1.20
**Pack-years**	<20 (ref.)	7.7	5.4–10.7	1	
	≥20	4.6	3.4–6.3	**0.45**	**0.21–0.97**
**Time-period of PATH Study**	2014/15-2015/16 (W2-W3) (ref.)	5.2	3.2–8.5	1	
	2015/16-2016/17 (W3-W4)	4.2	2.8–6.3	0.83	0.45–1.53
	2016/17-2018/19 (W4-W5)	7.5	4.7–11.7	1.46	0.69–3.09
	2018/19–2021 (W5-W6)	6.5	4.5–9.3	1.48	0.68–3.24
**Interview mode** ^d^	In-person (ref.)	6.0	4.7–7.8	1	
	Telephone	5.8	3.7–8.9	0.67	0.32–1.40

* Cigarette discontinuation at follow-up: daily smoking → no smoking vs any smoking. Data were aggregated across the study period and analyzed as wave pairs using GEE (generalized estimating equations) modeling. AOR: adjusted odds ratio. Percentages, 95% CIs and AORs are weighted. PATH: Population Assessment of Tobacco and Health. W: Wave. GED: Generalized Equivalency Diploma. ACASI: audio computer-assisted self-interview.

a Ever diagnosed with congestive heart failure, a stroke, or a heart attack.

b Ever diagnosed with chronic obstructive pulmonary disease, chronic bronchitis, or emphysema.

c Ever diagnosed with congestive heart failure, a stroke, a heart attack, chronic obstructive pulmonary disease, chronic bronchitis, or emphysema.

d Interview mode variable from follow-up wave was used rather than from baseline wave due to W6 data collection using both ACASI in-person interviews and telephone interviews.

# Excluded from adjusted analysis due to collinearity with other variables. Bold font indicates statistical significance at alpha=0.05.

### Predictors of cigarette reduction among adults with no plans to ever quit, who did not discontinue cigarette smoking

Among adults who at baseline were aged ≥21 years, smoked cigarettes every day, did not use e-cigarettes, had no plans to ever quit cigarette smoking, and who at follow-up did not discontinue cigarette smoking (n=2223 observations from 1228 individuals), 5.2% (95% CI: 4.0–6.6) (Table 3) reduced to non-daily cigarette smoking at follow-up. Those with higher nicotine dependence had lower odds of reducing cigarette smoking than those with lower dependence (2.3% vs 6.7%, AOR=0.48; 95% CI: 0.25–0.90) (Table 3), females had lower odds of reducing cigarette smoking than males (3.4% vs 6.6%, AOR=0.52; 95% CI: 0.32–0.85), older age groups (aged ≥40 years) had lower odds of reducing cigarette smoking than younger age groups, and non-Hispanic White people had lower odds of reducing cigarette smoking than non-Hispanic Black people (3.5% vs 12.2%). Cigarette reduction rates were also lower in 2014/15–2015/16 compared to the time period 2018/19–2021 (4.1% vs 6.9%).

**Table 3:** Predictors of cigarette reduction* among adults in the United States who at baseline were aged ≥21 years, smoked cigarettes daily, had no plans to ever quit smoking, did not use e-cigarettes, and who at follow-up did not discontinue smoking, Population Assessment of Tobacco and Health (PATH) study, 2014/15–2021 (N=2223 observations from N=1228 individuals)

Characteristics	Categories	%	95% **CI**	AOR	95% **CI**
**Overall**		5.2	4.0–6.6		
**Age** (years)	21–24	12.4	7.3–20.4	**5.36**	**2.48–11.57**
	25–39	8.3	5.4–12.5	**2.92**	**1.51–5.66**
	40–54	3.9	2.5–6.2	1.49	0.78–2.82
	55–69 (ref.)	2.8	1.9–4.3	1	
	≥70	5.2	2.5–10.5	1.74	0.73–4.17
**Sex**	Male (ref.)	6.6	4.8–8.9	1	
	Female	3.4	2.5–4.7	**0.52**	**0.32–0.85**
**Race/Ethnicity**	Non-Hispanic White (ref.)	3.5	2.4–5.0	1	
	Non-Hispanic Black	12.2	8.4–17.5	**2.61**	**1.45–4.70**
	Non-Hispanic, other race including multi-racial	3.5	1.3–9.5	0.53	0.13–2.14
	Hispanic	10.8	6.8–16.8	1.78	0.81–3.91
**Household income** ($)	<75000 (ref.)	5.1	4.0–6.6	1	
	≥75000	4.6	1.6–12.6	1.29	0.42–3.98
	Not reported	6.4	3.1–12.7	1.22	0.50–3.00
**Education level**	Less than high school/GED (ref.)	5.5	3.8–7.9	1	
	High school graduate	4.4	2.8–6.7	0.86	0.50–1.49
	Some college/associate	6.4	4.1–9.8	1.59	0.83–3.04
	Bachelor’s degree or higher	2.5	0.6–9.0	0.70	0.08–5.72
**Ever diagnosed with heart condition^a^**	No	5.2	4.0–6.8	^#^	
	Yes	4.4	2.1–8.8		
**Ever diagnosed with lung condition^b^**	No	5.4	4.2–7.0	^#^	
	Yes	4.0	2.4–6.7		
**Ever diagnosed with heart or lung condition^c^**	No	5.4	4.1–7.1	^#^	
	Yes	4.4	2.8–6.9		
**Past year quit attempt**	No (ref.)	4.9	3.8–6.3	1	
	Yes	8.1	4.7–13.6	1.32	0.71–2.48
**Interest in quitting**	None (ref.)	5.5	3.7–8.2	1	
	Low	4.3	2.9–6.2	0.73	0.41–1.29
	Moderate/high	5.7	3.9–8.2	0.93	0.51–1.68
**Perceived addiction**	Not at all addicted (ref.)	13.5	8.2–21.4	1	
	Somewhat addicted	6.0	4.4–8.0	0.63	0.28–1.41
	Very addicted	2.7	1.6–4.5	0.44	0.191.01
**Heaviness of Smoking Index** (HSI)	0–3 (ref.)	6.7	5.2–8.7	1	
	4–6	2.3	1.2–4.2	**0.48**	**0.25–0.90**
**Pack-years**	<20	9.5	7.2–12.3	^#^	
	≥20	1.7	1.1–2.6		
**Time-period of PATH Study**	2014/15-2015/16 (W2-W3) (ref.)	4.1	2.3–7.3	1	
	2015/16-2016/17 (W3-W4)	4.1	2.6–6.2	1.13	0.58–2.21
	2016/17-2018/19 (W4-W5)	5.1	3.2–7.9	1.66	0.75–3.64
	2018/19–2021 (W5-W6)	6.9	5.2–9.0	**2.32**	**1.01–5.36**
**Interview mode^d^**	In-person (ref.)	4.8	3.6–6.3	1	
	Telephone	7.1	5.1–9.9	0.87	0.42–1.82

* Cigarette consumption reduction at follow-up: daily smoking → non-daily smoking vs daily smoking. Data were aggregated across the study period and analyzed as wave pairs using GEE (generalized estimating equations) modeling. AOR: adjusted odds ratio. Percentages, 95% CIs and AORs are weighted. n= 2223 observations in the unadjusted analyses (i.e. percentages) and n=2305 observations in the adjusted analyses (i.e. AORs). The n is larger in the adjusted analyses because fewer variables were required to have complete data in the adjusted analyses (see # below). PATH: Population Assessment of Tobacco and Health. W: Wave. GED: Generalized Equivalency Diploma. ACASI: audio computer-assisted self-interview.

a Ever diagnosed with congestive heart failure, a stroke, or a heart attack.

b Ever diagnosed with chronic obstructive pulmonary disease, chronic bronchitis, or emphysema.

c Ever diagnosed with congestive heart failure, a stroke, a heart attack, chronic obstructive pulmonary disease, chronic bronchitis, or emphysema.

d Interview mode variable from follow-up wave was used rather than from baseline wave due to W6 data collection using both ACASI in-person interviews and telephone interviews.

# Excluded from adjusted analysis due to collinearity with other variables. Bold font indicates statistical significance at alpha=0.05.

### E-cigarette uptake and cigarette discontinuation or reduction among adults with no plans to ever quit

Among adults who at baseline were aged ≥21 years, smoked cigarettes every day, did not use e-cigarettes, and had no plans to ever quit cigarette smoking (11.1%; 95% CI: 9.3–13.2) discontinued or reduced cigarette smoking at follow-up (Table 4). While only 2% took up daily e-cigarette use, those who did experienced a 53.5% (95% CI: 32.6–73.2) cigarette discontinuation or reduction rate, while those who did not take up any e-cigarette use experienced a 10.0% (95% CI: 8.3–12.0) cigarette discontinuation or reduction rate (AOR=14.58; 95% CI: 5.99–35.50). There was no difference in cigarette discontinuation or reduction rates between those who took up non-daily e-cigarette use (9.8%; 95% CI: 5.2–17.8) and those who did not take up any e-cigarette use (10.0%; 95% CI: 8.3–12.0; AOR=1.10; 95% CI: 0.49–2.46).

**Table 4:** E-cigarette uptake and cigarette discontinuation* or reduction at follow-up among adults in the United States who at baseline were aged ≥21 years, smoked cigarettes daily, had no plans to ever quit smoking, and did not use e-cigarettes, Population Assessment of Tobacco and Health (PATH) Study, 2014/15–2021 (N=2440 observations from N=1369 individuals)

Population	E-cigarette uptake	N denominator	%	95% CI	AOR^a^	95% CI
All	Overall	2440	11.1	9.3–13.2		
	No uptake (ref.)	2234	10.0	8.3–12.0	1	
	Non-daily uptake	147	9.8	5.2–17.8	1.10	0.49–2.46
	Daily uptake	59	53.5	32.6–73.2	**14.58**	**5.99–35.50**

Population	E-cigarette characteristic	N denominator	%	**95% CI**	AOR^b^	**95% CI**
Those who took up e-cigarette use (N=169 for adjusted analyses)	Overall	172^c^	24.2	15.1–36.4		
	Tobacco flavor (ref.)	38	12.7	3.7–35.5	1	
	Menthol/mint flavor	20	20.0	6.0–49.6	0.82	0.03–26.56
	Fruit flavor	59	18.3	8.634.6	1.12	0.05–23.08
	Combination flavor	55	42.2	25.3–61.1	2.68	0.12–62.13
	Disposable device (ref.)	50	28.4	15.8–45.5	1	
	Cartridge device	51	9.9^†^	3.7–23.9	0.25	0.01–5.79
	Tank device	71	32.6	19.7–48.9	1.39	0.24–7.80
	Non-daily uptake (ref.)	113	5.6	2.5–12.2	1	
	Daily uptake	59	54.1	33.5–73.3	**25.29**	**2.22–288.26**

* Cigarette discontinuation or reduction at follow-up: daily smoking → no smoking or nondaily smoking vs daily smoking. Sample sizes (n) are unweighted. Percentages, AORs, and 95% CIs are weighted. AOR: Adjusted odds ratio.

a Analyses were adjusted for age, sex, race/ethnicity, household income, HSI, and time-period assessed at baseline, and for interview mode assessed at follow-up.

b Analyses were adjusted for age, sex, race/ethnicity, income, HSI, and time-period assessed at baseline, and for interview mode, e-cigarette flavor, e-cigarette device type, and frequency of e-cigarette use assessed at follow-up. N denominator for adjusted analyses =169 (3 missing income observations dropped due to collinearity).

c n=34 missing data on e-cigarette flavor or e-cigarette device type.

† Estimate should be interpreted with extra caution because it has low statistical precision. It is based on a denominator sample size of <50, or the coefficient of variation of the estimate or its complement is larger than 30%. Bold font indicates statistical significance at alpha=0.05.

Among those adults who took up any e-cigarette use, those who used e-cigarettes daily had higher odds of cigarette discontinuation or reduction than those who used e-cigarettes non-daily (AOR=25.29; 95% CI: 2.22–288.26, n=169 [3 missing income observations dropped due to collinearity]) (Table 4). Neither e-cigarette flavor nor e-cigarette device type was significantly associated with cigarette discontinuation or reduction when controlling for other variables, including e-cigarette use frequency (Table 4).

## Discussion

In this US nationally representative study of adults aged ≥21 years who smoked cigarettes daily and had no plans to ever quit smoking, and among whom one-quarter had been diagnosed with a heart or lung condition, lower income, and higher pack-years, were associated with lower rates of cigarette discontinuation. Among those who did not discontinue cigarette smoking, lower nicotine dependence, younger age, male sex, Non-Hispanic Black race (vs Non-Hispanic White race), and assessment in 2018/19–2021 (vs 2014/15–2015/16), were associated with higher rates of cigarette reduction. It is important to note that the more recent assessments span a longer observation period (2–3 years) compared to the earlier assessments (1 year), which might influence the observed rates of reduction as people have more time to reduce their cigarette smoking over longer observation periods. Uptake of daily e-cigarette use was low (2%) but strongly associated with cigarette discontinuation or reduction compared to no uptake or non-daily uptake of e-cigarette use. Neither e-cigarette flavor nor e-cigarette device type was associated with cigarette discontinuation or reduction, though sample size was limited due to very low uptake of e-cigarette use among adults who did not plan to ever quit, consistent with e-cigarette uptake being more common among youth and younger adults^28^.

Findings from this study yield several implications and directions for future research. First, given the persistence of a consistently sized proportion of people who smoke cigarettes, smoke them daily and do not plan to quit^1,4^, we found an increasing trend between 2014 and 2021 in reducing cigarette smoking (without discontinuing smoking) among this proportion of people. Further research with subsequent waves of data can investigate whether such reductions to non-daily smoking will eventually lead to complete smoking discontinuation and subsequent long-term cessation, or whether non-daily smoking persists or the individual reverts to daily smoking and for which people. Recent research using PATH Study data among the broader population of all adults who smoked cigarettes indicated a linear increase in cigarette discontinuation rates between 2014–2021^29^, which we did not observe here among adults aged ≥21 years who smoked cigarettes daily and had no plans to quit. However, our findings suggest there may have been an increase since 2018 in reducing cigarette smoking among the group of people who did not plan to ever quit smoking, observed during a period of various tobacco control actions and market changes occurring in the US^15,30^, including the growth in the market of e-liquids made with nicotine salts, which enable more efficient nicotine delivery and which may facilitate switching form cigarette smoking^14^. Although not evaluated here, studies have shown that differences in reducing from daily to non-daily smoking are associated with differences/reductions in nicotine dependence and biomarkers of exposure to harmful and potentially harmful constituents^30-33^, which in turn may positively impact health.

Finally, evidence from randomized clinical trials on the effectiveness of nicotine-containing e-cigarettes for cigarette smoking cessation continues to grow^34,35^, though such studies tend not to enroll people diagnosed with heart or lung conditions or who do not plan to ever quit smoking. A recent randomized naturalistic study that included people who did not want to quit smoking found that unguided uptake of e-cigarette use was associated with cigarette cessation^7^. Our study notably adds to the literature in its refinement of the population of interest and inclusion of those often excluded from intervention studies.

### Limitations

Despite the large size of the PATH Study data set, we focused on an understudied subset of the population and limitations of our study include having relatively small sample sizes of people who took up daily e-cigarette use, which limited our statistical power to identify associations between e-cigarette flavor, device type, and cigarette discontinuation or reduction. Second, we did not evaluate why people who did not plan to quit smoking later took up e-cigarette use (e.g. curiosity/experimentation, smoking restrictions, encouragement by social networks, changes in harm perceptions, later decided to try to quit, etc). Also, the 2021 assessment occurred during the COVID-19 pandemic, at which time PATH Study interviews were conducted both in-person and via telephone^18^, and differences in cigarette discontinuation rates by interview mode have been identified among the general population of adults who smoke cigarettes^29^. We adjusted our analyses for interview mode though it was not significantly related to our outcomes. Nonetheless, findings using data from 2021 should be interpreted with caution. We were also unable to account for residual confounding, including being unable to account for e-cigarette use and other time-limited behaviors occurring between wave-to-wave assessments, and thus our findings do not distinguish between whether e-cigarette uptake occurred before or after cigarette smoking discontinuation or reduction. Additionally, self-reported data may introduce information bias and misclassification into our study. Lastly, our analyses were among a group of people who smoked cigarettes daily and were initially not using e-cigarettes; thus, our findings are not generalizable to the broader population of people who use both cigarettes and e-cigarettes.

## Conclusions

Our findings of an increasing trend in rates of reducing or discontinuing cigarette smoking among people who smoked daily and did not plan to ever quit smoking, suggest a recent change among this segment of people who smoke. Our findings of a strong association between daily e-cigarette uptake and cigarette reduction/discontinuation among a segment of the population at high risk of smoking-attributable morbidity and mortality, add to the growing evidence on the role of e-cigarettes in moving adult smokers down the continuum of risk.
