# Supplementary material for: Predictors of cigarette discontinuation and reduction among adults never planning to quit smoking: Longitudinal findings from the U.S. Population Assessment of Tobacco and Health (PATH) study, 2014/15–2021
Source: Tob Prev Cessat. 2026 Sep 30;12:10.18332/tpc/225322. doi: 10.18332/tpc/225322 (PMC13628602; doi:10.18332/tpc/225322)
Supplement: Supplementary file 1 [file TPC-12-56-s001.pdf]

TOBACCO PREVENTION AND CESSATION

Supplementary file

© 2026 Kasza K.A. et al.

DOI:
10.18332/tpc/225322

The content has been provided by the author(s) and has not been reviewed, verified, or endorsed by European Publishing. It may not have undergone peer review. The views, opinions, and recommendations expressed are solely those of the author(s) and do not necessarily reflect the position of European Publishing. European Publishing accepts no responsibility or liability for any consequences arising from the use of, or reliance on, this content.

Supplemental Table 1. Population Assessment of Tobacco and Health (PATH) study measures

Measures	Categorizations	Questions and Responses Used in Categorizations
**Sociodemographic characteristics, health status, and interview mode** (assessed at baseline wave of each wave pair)		
Age (years)	(1) 21-24<br>(2) 25-39<br>(3) 40-54<br>(4) 55-69<br>(5) 70+	"What is your age?"
Sex	(1) Male<br>(2) Female	"What is your sex?" with response options: male/ female
Race/Ethnicity	(1) Non-Hispanic, White<br>(2) Non-Hispanic, Black<br>(3) Non-Hispanic, Other race including Multi-Racial<br>(4) Hispanic	"Are you Hispanic, [Latino I Latina I Latino or Latina], or of Spanish origin? Choose all that apply." with response options No, not of Hispanic, [Latino I Latina I Latino or Latina], or Spanish origin/Yes, Mexican, Mexican American, [Chicano I Chicana I Chicano or Chicana]/Yes, Puerto Rican/Yes, Cuban/Yes, another Hispanic, [Latino I Latina I Latino or Latina], or Spanish origin<br><br>"What is your race? Choose all that apply." with response options: White/Black or African American/American Indian or Alaska Native/Asian Indian/Chinese/Filipino/Japanese/Korean/Vietnamese/Other Asian/Native Hawaiian/Guamanian or Chamorro/Samoan/Other Pacific Islander
Household Income	(1) <$75000<br>(2) $75000+<br>(3) Not reported	"Which of the following categories best describes your total **household** income in the past 12 months? This is the total income before taxes of all persons in your household combined. Please include money from jobs, relatives, pensions, dividends, interest, social security payments or retirement benefits, net income from business, farm or rent, and any other money received by household members." with response options: Less than $10,000/$10,000 to $14,999/$15,000 to $24,999/$25,000 to $34,999/$35,000 to $49,999/$50,000 to $74,999/$75,000 to $99,999/$100,000 to $149,999/$150,000 to $199,999/$200,000 or more
Education	(1) Less than High School/GED<br>(2) High School graduate<br>(3) Some college/associate<br>(4) Bachelor's degree+	"What is the highest grade or level of school you have completed?" with response options: Less than high school/Some high school, no diploma/GED/High school graduate—diploma/Some college but no degree/Associate degree—occupational/vocational/Associate degree—academic program/Bachelor's degree (ex: BA, AB, BS)/Master's degree (ex: MA, MS, MEng, MEd, MSW)/Professional school degree (ex: MD, DDS, DVM, JD)/Doctorate degree (ex: PhD, EdD)
Heart condition	(0) No<br>(1) Yes	"Has a doctor, nurse or other health professional ever told you that you had any of the following heart conditions? Choose all that apply." We included: Congestive heart

		failure/A stroke/A heart attack (also called myocardial infarction) or needed bypass surgery
Lung condition	(0) No<br>(1) Yes	"Has a doctor, nurse or other health professional ever told you that you had any of the following lung or respiratory conditions? Choose all that apply." We included: COPD/Chronic bronchitis/Emphysema"
Interview mode	(1) In-Person<br>(2) Telephone	Not applicable
**Analytic sample-defining measures** (assessed at baseline wave of each wave pair)		
Daily cigarette smoking	Smokes cigarettes every day	"Do you now smoke cigarettes…" with response options: every day/some days/not at all
No plans to ever quit	Does not plan to ever quit cigarettes/tobacco[a] for good	"Do you plan to ever quit [cigarettes/tobacco][a] for good?" with response options: yes/no
No e-cigarette use	Does not use e-cigarettes at all	"Do you now use [e-cigarettes][b]…" with response options: every day/some days/not at all
**Smoking-related measures that may be related to cigarette discontinuation or reduction** (assessed at baseline wave of each wave pair)		
Made a quit attempt in previous year	(0) Have not made a quit attempt in past year<br>(1) Have made a quit attempt in past year	"In the past 12 months, have you tried to quit [smoking cigarettes/using tobacco] completely?"
Interest in quitting	(0) None (scale 1)<br>(1) Low (scale 2-4)<br>(2) Moderate/high (scale 5-10)	"Overall, on a scale from 1 to 10 where 1 is not at all interested and 10 is extremely interested, how interested are you in quitting [cigarettes/tobacco][a]? Please choose a number from 1 to 10."[c]
Perceived addiction to smoking	(1) Not at all addicted<br>(2) Somewhat addicted<br>(3) Very addicted	"Do you consider yourself addicted to [cigarettes/tobacco][a]?" with response options: No, not at all; Yes, somewhat addicted; Yes, very addicted
Dependence: Heaviness of smoking index (HSI)	(0) HSI 0-3<br>(1) HSI 4-6	"On average, about how many cigarettes do you now smoke each day? A pack usually has 20 cigarettes in it" and "How soon after you wake up do you typically smoke your first cigarette of the day? Please enter the number of minutes or hours."[d]
Pack-years of cigarette smoking	(0) Less than 20 years<br>(1) 20+ years	"About how long [have you been smoking/did you smoke] fairly regularly? Do not count any time you may have stayed off cigarettes." "On average, about how many cigarettes did you smoke per day while you were smoking fairly regularly?"[e]
**E-cigarette uptake measures** (assessed at follow-up wave among those who did not use e-cigarettes at baseline wave of each wave pair)		
E-cigarette use frequency	(1) No e-cigarette use<br>(2) Nondaily e-cigarette use<br>(3) Daily e-cigarette use	"Do you now use [e-cigarettes][c]…" with response options: every day/some days/not at all
E-cigarette flavor	(1) Tobacco flavor only<br>(2) Menthol/mint flavor only<br>(3) Fruit/sweet/other flavor only	"In the past 30 days, which flavors of [e-cigarettes] have you used? Choose all that apply" with response options: Tobacco-flavored; Menthol or mint; Clove or spice; Fruit; Chocolate; An alcoholic drink (such as wine, cognac, margarita, or other cocktails); A non-alcoholic drink (such as

	(4) Any combination of above flavor categories	coffee, soda, energy drinks, or other beverages); Candy, desserts, or other sweets; Some other flavor
E-cigarette device type	(1) Disposable<br>(2) Cartridge<br>(3) Tank/mod	“For the following question, please think about the electronic nicotine product you use most often. What kind of electronic nicotine product is it?” with response options: A disposable device; A device that uses replaceable prefilled cartridges; A device with a tank that you refill with liquids; A mod system; Something else
**Outcome measures** (assessed at follow-up wave among those who smoked cigarettes daily at baseline wave of each wave pair)		
Cigarette discontinuation	(1) Cigarette discontinuation: Is currently smoking cigarettes “not at all,” or responded “no” to smoking cigarettes in the past 12 months<br>(2) Cigarette smoking: Is currently smoking “every day” or “some days”	
Cigarette reduction among those who did not discontinue cigarette use	(1) Cigarette reduction: Is currently smoking “some days”<br>(2) Daily cigarette smoking: Is currently smoking “every day”	"In the past 12 months, have you smoked a cigarette, even one or two puffs?" with response options: yes/no<br>"Do you now smoke cigarettes…” with response options: every day/some days/not at all
Cigarette discontinuation or reduction	(1) Cigarette discontinuation or reduction: Is currently smoking cigarettes “some days” or “not at all,” or responded “no” to smoking cigarettes in the past 12 months<br>(2) Daily cigarette smoking: Is currently smoking “every day”	

[a]Those who smoked cigarettes and who used any other non-e-cigarette tobacco product and had ever used that tobacco product ‘fairly regularly’ were asked about intending to quit using "tobacco" rather than specifically about intending to quit smoking "cigarettes."
[b]“E-cigarettes” refers to all e-products (i.e., e-cigarettes, e-cigars, e-pipes and e-hookah).
[c]We include interest in quitting even though our sample is limited to those with no plans to ever quit because people may have interest in quitting even if they do not have plans to quit.
[d]Based on Fagerström (Fagerström, 1978) (i.e., summed categorized cigarettes smoked per day [where 0=10 or less, 1=greater than 10 and less than 21, 2=greater than 20 and less than 31, and 4= greater than 30] with categorized minutes to first cigarette after waking [where 0=greater than 60 minutes of waking, 1=greater than 30 minutes of waking and less than or equal to 60 minutes of waking, 2= greater than or equal to 6 minutes of waking and less than or equal to 30 minutes of waking, 3= within 5 minutes of waking]).
[e]Calculated as number of cigarettes smoked per day multiplied by number of years smoked fairly regularly.
